# Transpulmonary Plasma Endothelin-1 Arterial:Venous Ratio Differentiates Survivors from Non-Survivors in Critically Ill Patients with COVID-19-Induced Acute Respiratory Distress Syndrome

**DOI:** 10.3390/ijms251910640

**Published:** 2024-10-02

**Authors:** Alice G. Vassiliou, Anastasia Roumpaki, Chrysi Keskinidou, Nikolaos Athanasiou, Stamatios Tsipilis, Edison Jahaj, Charikleia S. Vrettou, Vassiliki Giannopoulou, Asimenia Halioti, Georgios Ferentinos, Ioanna Dimopoulou, Anastasia Kotanidou, David Langleben, Stylianos E. Orfanos

**Affiliations:** 1 First Department of Critical Care Medicine & Pulmonary Services, School of Medicine, National & Kapodistrian University of Athens, “Evangelismos” Hospital, 10676 Athens, Greece; ana_roumpaki@yahoo.com (A.R.); chrysakes29@gmail.com (C.K.); nikolaosathanasiou14@gmail.com (N.A.); stamostsipil@gmail.com (S.T.); edison.jahaj@gmail.com (E.J.); vrettou@hotmail.com (C.S.V.); vaso.giannop88@gmail.com (V.G.); minaxal@yahoo.gr (A.H.); drgeorgeferentinos@hotmail.gr (G.F.); idimo@otenet.gr (I.D.); akotanid@gmail.com (A.K.); 2 Center for Pulmonary Vascular Disease, Division of Cardiology, Azrieli Heart Center and Lady Davis Institute for Medical Research, Jewish General Hospital, McGill University, Montreal, QC H3T 1E2, Canada; david.langleben@mcgill.ca

**Keywords:** endothelin-1, COVID-19, ARDS, clearance, prognosis

## Abstract

Endothelin-1 (ET-1) is a potent vasoconstrictor produced by endothelial cells and cleared from circulating blood mainly in the pulmonary vasculature. In a healthy pulmonary circulation, the rate of local production of ET-1 is less than its rate of clearance. In the present study, we aimed to investigate whether the abnormal pulmonary circulatory handling of ET-1 relates to poor clinical outcomes in patients with coronavirus disease 2019 (COVID-19)-induced acute respiratory distress syndrome (ARDS). To this end, central venous and systemic arterial ET-1 plasma levels were simultaneously measured on Days 1 and 3 following ICU admission in mechanically ventilated COVID-19 patients with ARDS (COVID-19 ARDS, N = 18). Central venous and systemic arterial ET-1 plasma levels were also measured in two distinct SARS-CoV-2-negative mechanically ventilated critically ill patient groups, matched for age, sex, and critical illness severity, with ARDS (non-COVID-19 ARDS, N = 14) or without ARDS (non-COVID-19 non-ARDS, N = 20). Upon ICU admission, COVID-19-induced ARDS patients had higher systemic arterial and central venous ET-1 levels compared to the non-COVID-19 ARDS and non-COVID-19 non-ARDS patients (*p* < 0.05), yet a normal systemic arterial:central venous (A:V) ET-1 ratio [0.63 (0.49–1.02)], suggesting that pulmonary ET-1 clearance is intact in these patients. On the other hand, the non-COVID-19 ARDS patients demonstrated abnormal ET-1 handling [A:V ET-1 ratio 1.06 (0.93–1.20)], while the non-COVID-19 non-ARDS group showed normal ET-1 handling [0.79 (0.52–1.11)]. On Day 3, the A:V ratio in all three groups was <1. When the COVID-19 ARDS patients were divided based on 28-day ICU mortality, while their systemic arterial and central venous levels did not differ, the A:V ET-1 ratio was statistically significantly higher upon ICU admission in the non-survivors [0.95 (0.78–1.34)] compared to the survivors [0.57 (0.48–0.92), *p* = 0.027]. Our results highlight the potential importance of ET-1 as both a biomarker and a therapeutic target in critically ill COVID-19 patients. The elevated A:V ET-1 ratio in non-survivors suggests that the early disruption of pulmonary ET-1 handling may be a key marker of poor prognosis.

## 1. Introduction

Infection by the severe acute respiratory syndrome coronavirus 2 (SARS-CoV-2) is a major cause of morbidity and mortality and resulted in a severe pandemic [1,2,3]. The virus attaches to the angiotensin-converting enzyme (ACE)-2, which is found as an ectoenzyme on arterial, venous, and capillary endothelial cells. In addition to injuring the endothelium, leading to many types of vascular complications, the subsequent corporeal inflammatory reaction magnifies the injury and the severity of the systemic illness [4]. The lung is particularly affected, possibly because the major method of transmission of the virus is via inhalation, but the lung circulation is also particularly rich in ACE-2. Affected patients may have a mild respiratory illness, or they may progress to severe lung injury and acute respiratory distress syndrome (ARDS), which carries a high mortality [2]. Several studies have shown that the pathophysiology of coronavirus disease 2019 (COVID-19)-induced ARDS is more complex and diverse compared to typical ARDS, which is characterized by “reduced lung volume and decreased compliance” [5,6,7,8].

Endothelial cells are key factors in COVID-19 disease establishment and progression. The SARS-CoV-2-infected cells become activated, leading to the establishment of vascular dysfunction, inflammation, and a hypercoagulable status [9]. The vascular tone is also affected; endothelial dysfunction often results in decreased nitric oxide (NO) synthesis and the increased release of vasoconstrictors, including endothelin-1 (ET-1) and angiotensin II [10,11]. Endothelin-1 is a potent vasoconstrictor produced by endothelial cells throughout the body. Circulating plasma ET-1 levels are generally low (0.4–2.0 pg/mL), but they rise in disease states, including cardiovascular diseases, cancer, chronic pain, and asthma [12]. The major site of circulating endothelin clearance is in the lung circulation, via endothelial endothelin type B (ETB) receptors [13,14]. Normally, the plasma ET-1 ratio for blood entering the lung compared to that leaving the lung appears to be equal to unity [14]. This occurs since approximately 50% of circulating ET-1 is removed by the human lung; however, a comparable amount is released back into circulation by the lung, explaining the absence of an arteriovenous ET-1 gradient across the pulmonary circulation [15]. In healthy control subjects, the plasma ET-1 ratio has been found to be 0.59–0.7 [16,17]. Decreased clearance of ET-1 has been described in pulmonary hypertension, pulmonary fibrosis, coronary disease, asthma, ARDS, septic shock, and collagen vascular disorders such as systemic sclerosis and systemic lupus erythematosus, among others [16,17,18,19,20,21,22,23,24,25,26,27].

In addition to its vasoconstrictive action, ET-1 is a potent mitogen for smooth muscle cells and fibroblasts, and ET-1-induced cytokine release causes the activation of the inflammatory cascade, leading to increased vascular permeability and eventually multi-organ failure [12,28,29,30].

Since pulmonary endothelial dysfunction is a hallmark of COVID-19-induced ARDS and considering that, until now, a small number of studies have reported elevated levels of ET-1 in the circulation of COVID-19 patients [31,32], in this study, we aimed to explore whether COVID-19 ARDS patients present with an imbalance between the pulmonary synthesis and clearance of ET-1. To this end, we measured central venous and systemic arterial ET-1 levels in COVID-19 ARDS patients to investigate whether the elevated circulating ΕΤ-1 levels previously reported are related to abnormal ET-1 clearance or net synthesis. Furthermore, we explored if any abnormal pulmonary ET-1 handling would reverse in COVID-19 ARDS patients who show clinical improvement.

## 2. Results

During the screening period, no patients were excluded based on the study’s criteria. Eventually, 18 COVID-19-induced ARDS Caucasian patients were enrolled with a median age of 73 (66–78) years and 72% were male. Upon ICU admission, the acute physiology and chronic health evaluation (APACHE) II score was 16 (10–21) and the sequential organ failure assessment (SOFA) score was 8 (6–9). Two mechanically ventilated critically ill control groups were also included; non-COVID-19 ARDS patients (N = 14) and non-COVID-19 non-ARDS patients (N = 20). Table 1 shows the demographics, clinical characteristics, biochemical, and laboratory data of the three patient groups upon ICU admission.

The patient groups were matched for age, sex, and critical illness severity scores. The non-COVID-19 ARDS patients had a respiratory infection (mostly caused by Gram-negative bacteria), whereas the non-COVID-19 non-ARDS patients were mainly surgical or trauma patients. As expected, the two ARDS groups had a lower PaO_2_/FiO_2_ ratio (*p* < 0.05), a higher respiratory rate (*p* < 0.05), and a higher positive end-expiratory pressure (PEEP) (*p* < 0.0001 and *p* < 0.05, respectively) compared to the non-ARDS patients. Regarding surrogate endothelial markers, plasma soluble intercellular adhesion molecule 1 (sICAM-1) levels were higher in the two groups of ARDS patients (*p* < 0.05), while sE-selectin levels were higher in the non-COVID-19 ARDS patients (*p* < 0.01).

**Figure 1:**
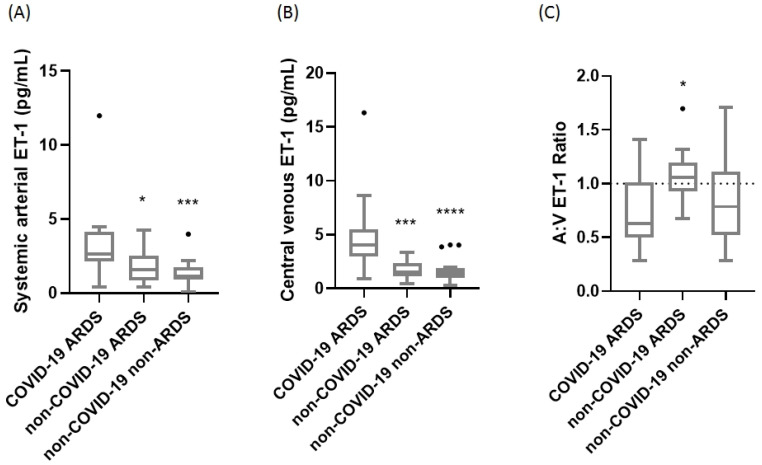
ICU admission ET-1 levels in the three critically ill groups. Systemic arterial (**A**) and central venous (**B**) ET-1 was measured in the COVID-19 ARDS (N = 18), non-COVID-19 ARDS (N = 14), and non-COVID-19 non-ARDS (N = 20) patients by enzyme-linked immunosorbent assay (ELISA). The systemic arterial:central venous (A:V) ET-1 ratio was calculated thereafter (**C**). The horizontal line represents an A:V ET-1 ratio of 1. Data are presented as box plots. Line in the middle of the box, median value; box edges, 25th to 75th centiles; whiskers, range of values; bullets, outliers. One-way ANOVA followed by Kruskal–Wallis was performed to test for differences between the groups. * *p* < 0.05, *** *p* < 0.01, **** *p* < 0.0001.

We then proceeded to concurrently measure the central venous and systemic arterial ET-1 levels in 18 COVID-19-induced ARDS patients, 14 non-COVID-19 ARDS patients, and 20 critically ill patients on Days 1 and 3 from ICU admission. COVID-19-induced ARDS patients had higher systemic arterial and central venous ET-1 levels upon ICU admission compared to the non-COVID-19 ARDS and the non-COVID-19 critically ill patients [2.66 (2.11–4.14) pg/mL vs. 1.59 (0.85–2.54) pg/mL and 1.12 (0.89–1.72) pg/mL, respectively] (Figure 1A; *p* < 0.05 and *p* < 0.001; respectively). Levels of central venous ET-1 were 4.04 (2.99–5.48) pg/mL vs. 1.52 (1.12–2.33) pg/mL and 1.40 (0.95–1.93) pg/mL, respectively (Figure 1B; *p* < 0.001 and *p* < 0.0001, respectively).

The systemic arterial:central venous (A:V) ratio was 0.63 (0.49–1.02) in our COVID-19-ARDS cohort, 1.06 (0.93–1.20) in the non-COVID-19 ARDS patients, and 0.79 (0.52–1.11) in the non-COVID-19 critically ill patients upon ICU admission (Figure 1C; *p* < 0.05).

**Figure 2:**
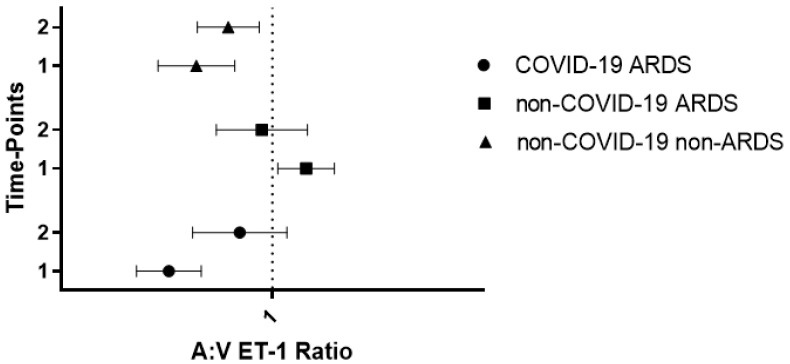
Systemic arterial:central venous (A:V) ET-1 ratio in the three critically ill groups at two time-points in the ICU. The A:V ET-1 ratio is shown upon ICU admission (time-point 1) in the COVID-19 ARDS (N = 18), non-COVID-19 ARDS (N = 14), and non-COVID-19 non-ARDS (N = 20) groups and on Day 3 after admission (time-point 2) in the COVID-19 ARDS patients (N = 10), the non-COVID-19 ARDS patients (N = 13), and the non-COVID-19 critically ill patients (N = 17). The vertical line represents an A:V ET-1 ratio of 1.

On Day 3 in the COVID-19 patients (N = 10), systemic arterial ET-1 levels remained unchanged as compared to admission [2.31 (1.78–4.60) pg/mL; *p* = 0.7], whereas central venous ET-1 levels decreased [2.62 (1.43–5.67) pg/mL; *p* = 0.4], albeit not statistically significantly. This resulted in a small rise in the A:V ratio compared to the ICU admission value [0.98 (0.60–1.17); *p* = 0.2]. In the two control groups, the A:V ET-1 ratios on Day 3 were 1.00 (0.69-1.16) in the non-COVID-19 ARDS patients (N = 13) and 0.89 (0.76–1.24) in the non-COVID-19 critically ill patients (N = 17) (*p* = 0.4 and *p* = 0.3, respectively, compared to their admission values). As opposed to ICU admission, on Day 3, the A:V ET-1 ratio was similar in the three groups studied (*p* = 0.9). Figure 2 shows the time progression of the A:V ET-1 ratio in the three groups.

The COVID-19-induced ARDS patient group was then subdivided into survivors (N = 12) and non-survivors (N = 5) according to 28-day ICU mortality. For one patient, we did not have the outcome since the patient was transferred to another facility on Day 20 from ICU admission. Table 2 lists the demographics, clinical characteristics, and laboratory data of survivors and non-survivors upon ICU admission. The two patient subgroups differed only in the respiratory rate. The critical illness severity scores, APACHE II and SOFA scores, and PaO_2_/FiO_2_ did not differ upon ICU admission between survivors and non-survivors.

**Figure 3:**
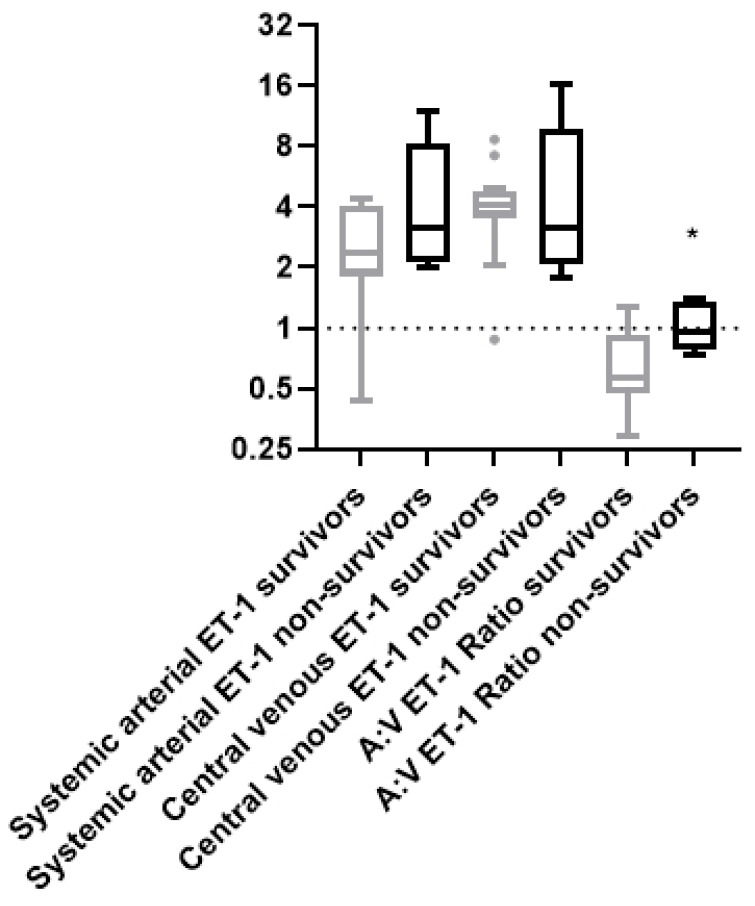
ICU admission ET-1 levels and ratio and 28-day ICU mortality. Systemic arterial, central venous, and A:V ET-1 ratio in the 28-day ICU survivors and non-survivors of COVID-19 ARDS. Systemic arterial and central venous ET-1 was measured in the COVID-19 ARDS survivors (N = 12) and non-survivors (N = 5) by enzyme-linked immunosorbent assay (ELISA). The systemic arterial:central venous (A:V) ET-1 ratio was calculated thereafter. The horizontal line represents an A:V ET-1 ratio of 1. Data are presented as box plots. Line in the middle of the box, median value; box edges, 25th to 75th centiles; whiskers, range of values; bullets, outliers. Grey box plots represent the survivors, and the black box plots the non-survivors. * *p* < 0.05 by Mann–Whitney.

The ET-1 systemic arterial and central venous levels did not differ between survivors and non-survivors. Systemic arterial levels were 2.37 (1.82–4.09) pg/mL vs. 3.18 (2.14–8.22) pg/mL, respectively (*p* = 0.3), and central venous levels were 4.10 (3.54–4.82) pg/mL vs. 3.17 (2.10–9.84) pg/mL, respectively, *p* = 0.3) (Figure 3). However, the A:V ET-1 ratio was higher upon ICU admission in the non-survivors [0.95 (0.78–1.34) vs. 0.57 (0.48–0.92), *p* = 0.027, Figure 3].

The Kaplan–Meier method was next used for survival probability estimation. The COVID-19 ARDS cohort was dichotomized above (high group) and below (low group) the median of A:V ET-1 ratio (0.63). The probability of mortality with time was significantly elevated in the high group. More specifically, the respective median time to mortality for the two aforementioned groups was 28 days [95% confidence interval (CI): 12–34] for the high group and 69 days (95% CI: 49–90) for the low group (log-rank test, *p* = 0.02).

**Figure 4:**
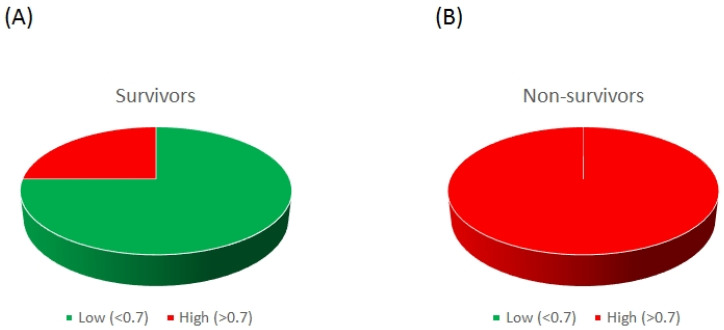
Distribution of the ICU admission A:V ET-1 ratio in COVID-19 ARDS survivors and non-survivors. Distribution of the A:V ET-1 ratio in COVID-19 ARDS survivors (**A**) and non-survivors (**B**). COVID-19 ARDS patients were assigned into two groups, low (green color) and high (red color), based on the cut-off value generated by the ROC curve (0.701). Distribution is shown as a 3D pie chart. *p* = 0.009 by the chi-square test.

A receiver operating characteristic (ROC) curve was subsequently generated to test the prognostic value of the A:V ET-1 ratio in 28-day ICU mortality. A cut-off of 0.701 showed a sensitivity of 100% and a specificity of 75%, generating an area under the curve (AUC) of 0.85 (0.66–1.00), *p* = 0.027. If the patients were assigned to high (above the cut-off) and low (below the cut-off) groups, 75% of the patients who eventually survived were in the low group, whereas 100% of the patients who died were in the high group (chi-square, *p* = 0.009; Figure 4).

**Figure 5:**
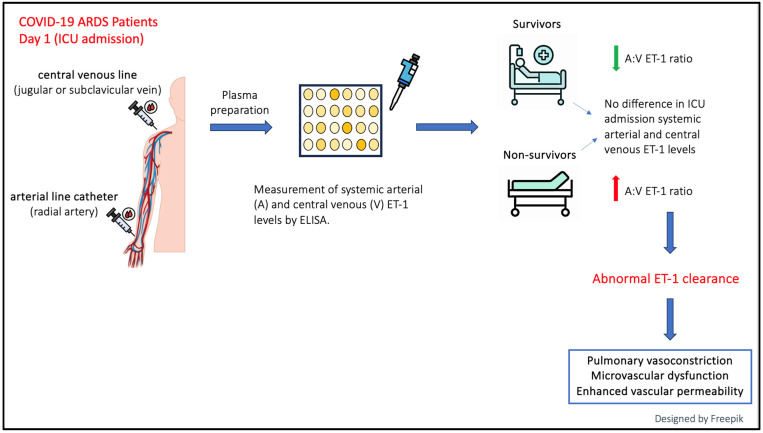
Summary of the research workflow and main findings. A simultaneous blood draw was performed in each COVID-19-induced ARDS patient from a central venous line (jugular or subclavicular vein) and from a peripheral arterial line catheter (radial artery). Endothelin-1 (ET-1) levels were measured in plasma blood samples by enzyme-linked immunosorbent assay (ELISA). Patients were divided based on 28-day ICU mortality. Although systemic arterial (A) and central venous (V) ET-1 levels did not differ in survivors and non-survivors, the A:V ET-1 ratio was statistically significantly higher upon ICU admission in the non-survivors compared to survivors, indicating net pulmonary ET-1 release. The abnormal clearance of ET-1 may lead to pulmonary vasoconstriction, microvascular dysfunction, and enhanced vascular permeability, which are considered the pathogenetic mechanisms of pulmonary hypertension and several related vascular complications. This figure has been designed using assets from “https://www.freepik.com/ (accessed on 12 September 2024)”.

Figure 5 outlines the workflow and key findings based on ET-1 measurements taken upon ICU admission.

## 3. Discussion

There has been extensive previous research on COVID-19 and lung injury, but few reports in COVID-19 infection focusing on ET-1 levels in venous blood samples [31,32,33,34,35]. However, to our knowledge, the effect of COVID-19 on transpulmonary ET-1 handling, and how abnormalities might relate to prognosis, has not been explored. This is the first study to measure central venous and systemic arterial ET-1 levels and hence measure both the synthesis and clearance of ET-1 in critically ill COVID-19-induced ARDS patients. We found that in these patients, both systemic arterial and central venous ET-1 levels were elevated; however, the A:V ratio was normal. Most interestingly, we were able to differentiate survivors and non-survivors among COVID-19-induced ARDS patients based on their A:V ET-1 ratio upon ICU admission.

Previous studies have shown increased plasma ET-1 levels in patients hospitalized with COVID-19 [31], while autoantibodies against the endothelin type A (ETA) receptor were found in COVID-19 patients with an unfavorable disease course [33]. Plasma ET-1 levels were significantly higher in acute COVID-19 compared to control subjects and were further elevated 3 months post-COVID-19 [34]. Blood plasma ET-1 levels were associated with an increased mortality risk during the acute phase of COVID-19, while the association of increased plasma ET-1 levels with COVID-19 mortality risk did not persist after 12 months [32]. In a randomized, double-blind, placebo-controlled trial, it was shown that the early administration of bosentan, a dual endothelin receptor antagonist (ERA), prevented disease progression and thromboembolic events in high-risk COVID-19 outpatients [35].

In the present study, we also found increased systemic arterial and central venous ET-1 levels in COVID-19-induced ARDS, further supporting the notion that it may be involved in the pathophysiology of severe COVID-19, consistent with its role as a potent vasoconstrictor and mediator of inflammation. In contrast to the study by Turgunova et al. [32], we were not able to find elevated central venous ET-1 levels in the non-survivors, possibly due to our small sample size. However, we were able to differentiate survivors and non-survivors among COVID-19-induced ARDS patients based on their A:V ET-1 ratio upon ICU admission. We showed that the ratio was higher upon ICU admission in patients who would eventually die, indicating net pulmonary ET-1 release. This release could reflect endothelial dysfunction or heightened inflammation in the lungs, contributing to the progression of ARDS and organ failure. Based on the ROC curve generated for 28-day mortality, the cut-off value for the A:V ET-1 ratio was 0.7. All patients who died had an A:V ratio > 0.7.

Three endothelin subtypes have been recognized, endothelin-1, 2, and 3, each with varying functions that depend on the receptor they bind [4]. Endothelin-1, a potent vasoconstrictor peptide produced by endothelial cells and degraded predominantly in the pulmonary vasculature, is considered a marker of lung injury. It functions via its binding to the endothelin type A and B receptors of the smooth muscle cells [36]. In general, the largest part of its contractile function acts through the ETA receptors, while in normal conditions, ET-1 is removed from the pulmonary circulation via the ETB receptors found on endothelial cells [37,38].

Endothelin-1 has been mostly studied in pulmonary arterial hypertension (PAH), a disease characterized mainly by tissue remodeling of the precapillary pulmonary vasculature, with subsequent right heart failure. In that disorder, ET-1 levels are high, mainly due to excess synthesis rather than reduced clearance [16,23,39,40,41,42,43,44,45,46,47,48,49,50,51,52].

Increased plasma ET-1 levels were found in ARDS patients compared to healthy control subjects [17,53] as a result of either increased synthesis or decreased clearance. These abnormalities reversed in the patients who recovered [17]. In critically ill patients with sepsis, including ARDS subjects, increased endothelin production contributed to local increases in vascular resistance, hypoperfusion, and the development of organ failure [54]. It has also been suggested that high circulating ET-1 levels may partly contribute to the development of pulmonary vasoconstriction and bronchoconstriction associated with acute respiratory failure [55]. Increased ET-1 immunohistochemical staining was seen in the lungs of subjects who died with ARDS [56], and it was demonstrated that in patients with ARDS, ET-1 is produced mainly in the lung and is associated not only with pulmonary vasoconstriction but also with the development of permeability edema, leading to the impairment of oxygenation [57]. Studies in animal models have also provided evidence for the involvement of ET-1 in lung injury and the use of endothelin receptor antagonists as potential treatments for human inflammatory lung diseases [58,59,60,61].

ET-1 levels may fluctuate in chronic pulmonary hypertension. In the setting of ARDS, the patients frequently present with acute pulmonary hypertension and hypoxemia. Studies indicate that ET-1 may contribute to hypoxia-induced pulmonary vasoconstriction [62,63]. Positive end-expiratory pressure and positive pressure ventilation may affect ET-1 levels. The two ARDS groups used had similar ventilator settings, suggesting that the differences observed in ET-1 levels may be due to the sequelae of the viral infection itself. Indeed, several studies have shown that the pathophysiology of ARDS caused by COVID-19 is more complex and diverse compared to typical ARDS of “reduced lung volume and decreased compliance” [5]. Moreover, published data indicate that the inflammatory profile in COVID-19 patients is different than that observed in patients with either ARDS or sepsis, with concentrations of inflammatory cytokines in both severely and critically ill COVID-19 patients being significantly lower even in those patients meeting the criteria for “hypo-inflammatory ARDS” [64,65,66,67,68,69,70].

As a surrogate of endothelial dysfunction, we also measured various soluble endothelial activation/damage indices. Our results showed an increase in sICAM-1 in COVID-19 and non-COVID-19 ARDS; however, we were not able to establish a correlation between the endothelial biomarker levels and ET-1 levels. We believe that this may be since ET-1 levels reflect the synthesis/clearance balance, which is mainly pulmonary, while other biomarker levels, measured in peripheral venous blood, might be affected by systemic vascular injury and inflammation.

While ET-1 is taken up and released by the pulmonary circulation, it is also metabolized by hepatocytes. However, this represents a minor component of ET-1 clearance, the great bulk of which is pulmonary. Since most of the central lines went through the superior vena cava, it is possible that not all blood sampled would adequately reflect venous return from the lower extremities or the portal circulation, and therefore, any hepatic effects might be missed. However, none of our patients had overt liver disease, and we hypothesize that its clearance is mainly by pulmonary circulation.

The limitations of our study must be mentioned. The main limitation was the small sample size but, despite that, we found significant differences among the clinical groups. To further assist in understanding our findings, we used two different critically ill cohorts, matched for age, sex, and critical illness severity, to validate our analysis of differences between the groups. Recruitment was hampered by the initial overwhelming patient load and resultant diversion of resources away from research, then by the reduced incidence of lung injury with the arrival of COVID-19 vaccines. Another limitation is that we did not collect swab samples from our patients to isolate and identify the particular variant. Finally, blood sampling in the COVID-19 patients occurred following dexamethasone administration, hence we could not measure cytokines to compare the inflammatory status of the patient groups. Even though COVID-19 has subsided, ICU admissions and mortality due to COVID-19 still exist, and our findings could be applicable to any future strain that becomes more virulent and causes fulminant lung injury, as was seen in the pre-vaccine era. Larger multi-center studies are warranted to validate our results and ensure the generalizability of the A:V ET-1 ratio as a prognostic marker.

## 4. Materials and Methods

This observational, case-control, single-center study included consecutive adult mechanically ventilated patients hospitalized in our intensive care unit (ICU, N = 18) with COVID-19-induced ARDS. Two groups matched for age, sex, and critical illness severity, as assessed by the APACHE II and SOFA scores, were also included as control groups: a group of SARS-CoV-2-negative mechanically ventilated non-ARDS patients (N = 20), mostly with central nervous system (CNS)-related injuries, and a group of SARS-CoV-2-negative mechanically ventilated ARDS patients (N = 14). SARS-CoV-2 infection was diagnosed by real-time reverse transcription PCR (RT-PCR) in nasopharyngeal swabs. The only exclusion criterion was the presence of acquired immunodeficiency syndrome. Based on a previous study on ET-1 clearance in ARDS [17], to detect a 40% increase in the A:V ET-1 ratio with a Cohen-d effect size of 0.9, an α-error probability < 0.05, and a power > 80%, it was estimated that a cohort of 16 critically ill patients and a group of 16 matched control subjects would suffice.

The enrolment took place between September 2021 to September 2023. The study was approved by the Hospital’s Research Ethics Committee (418/9-9-2021) and all procedures carried out on patients were in compliance with the Helsinki Declaration. Informed written consent was obtained from the patients’ next of kin.

Criteria for ARDS (Berlin definition) included PaO_2_/FiO_2_ < 300 mmHg and bilateral infiltrates in the chest X-ray [71]. Since ET-1 levels are known to fluctuate in patients with pulmonary hypertension [16], pre-existing pulmonary hypertension was recorded from the patient’s medical history. Patients with human immunodeficiency virus infection were excluded from the study. Demographics, vital signs, mechanical ventilation settings, arterial blood gases, APACHE II, SOFA scores, comorbidities, pharmaceutical treatment, and routine laboratory data were recorded for all patients enrolled in the study.

A simultaneous blood draw was performed in each patient from a central venous line (jugular or subclavicular vein) and from a peripheral arterial line catheter (radial artery) with a slow 3-min draw to avoid sample hemolysis upon ICU admission (within 24-h) and on Day 3 from admission, as has been described [16,17]. A second blood draw was not obtained if the patient was discharged earlier, was extubated, or if death occurred. The blood samples were collected in pre-chilled BD Vacutainer^®^ EDTA Tubes (Becton, Dickinson and Company, Franklin Lakes, NJ, USA), were centrifuged, and the resulting plasma was aliquoted and stored at −70 °C until use.

Endothelin-1 plasma levels were measured in the central venous and systemic arterial blood samples obtained upon ICU admission (within 24-h) and on Day 3 after admission by enzyme-linked immunosorbent assay (ELISA) (R&D Systems Inc., Minneapolis, MN, USA). The intra-assay coefficient of variability (CV) of the assay was 2.7% and the inter-assay CV was 6.3%, with a detection limit of 0.207 pg/mL. The researcher who performed the measurements was blinded to the samples measured.

Soluble (s)E-selectin (CV 5.8%, detection limit 0.027 ng/mL), soluble intercellular adhesion molecule 1 (sICAM-1) (CV 4.6%, detection limit 0.254 ng/mL), soluble vascular cell adhesion molecule-1 (sVCAM-1) (CV 3.1%, detection limit 1.26 ng/mL) (R&D Systems Inc., Minneapolis, MN, USA), and von Willebrand factor (vWf) (CV 5.2%, detection limit < 50 pg/mL, OriGene Technologies, Inc., Rockville, MD, USA) were measured in plasma samples by ELISA according to the manufacturers’ instructions. The assays use two different polyclonal antibodies against the molecules as catching and tagging antibodies. The researcher who performed the measurements was blinded to the samples measured.

Data are presented as individual values with percentages (N, %), mean ± standard deviation (SD) for normally distributed variables, or median with interquartile range (IQR) for variables with skewed distribution. Between-group comparisons were performed by the *t*-test or the non-parametric Mann–Whitney test, as appropriate. One-way ANOVA followed by Kruskal–Wallis was performed for more than two groups comparisons. Associations between qualitative variables were examined by the chi-square test. The Kaplan–Meier method was used for survival probability estimation and the log-rank test for two groups comparison. A ROC curve was plotted using 28-day ICU mortality as the classification variable and the A:V ET-1 ratio upon ICU admission as the prognostic variable. The optimal cut-off value for predicting 28-day mortality was calculated as the point with the greatest combined sensitivity and specificity. The analyses were performed with the IBM SPSS statistical package, version 22.0 (IBM Software Group, Armonk, NY, USA), and GraphPad Prism, version 8.0 (GraphPad Software, San Diego, CA, USA). All *p*-values are two-sided; *p* < 0.05 was considered significant.

## 5. Conclusions

In our COVID-19-induced ARDS cohort, we found high circulating plasma levels of ET-1 and a normal A:V ratio, suggesting a normal pulmonary ET-1 clearance. When COVID-19 patients were divided based on their 28-day ICU outcome, the A:V ET-1 ratio was higher in non-survivors compared to survivors, indicating net pulmonary ET-1 release. Thus, our study underscores the potential for the early measurement of pulmonary ET-1 handling to serve as a prognostic marker in critically ill COVID-19 patients. By identifying patients with impaired ET-1 handling early on, clinicians may be able to better stratify risk and potentially guide more targeted therapies. Further investigation into the role of ET-1 in ARDS pathophysiology, and its potential as a therapeutic target, could help improve outcomes in COVID-19.

## Figures and Tables

**Table 1:** Demographics, clinical characteristics, biochemical, and laboratory data of the 3 patient groups upon ICU admission.

Characteristics	COVID-19 ARDS	Non-COVID-19 ARDS	Non-COVID-19 Non-ARDS
Number of patients, N	18	14	20
Age (years)	73 (66–78)	64 (48–76)	62 (45–73)
Sex, N (%)			
Male	13 (72.2)	7 (50.0)	11 (55.0)
Female	5 (27.8)	7 (50.0)	9 (45.0)
Comorbidities, N (%)			
Hypertension	11 (61.1)	7 (50.0)	6 (30.0)
Dyslipidemia	4 (22.2)	4 (28.6)	5 (25.0)
Diabetes	4 (22.2)	6 (42.9)	3 (15.0)
Coronary disease	4 (22.2)	2 (14.3)	2 (10.0)
Cancer	3 (16.7)	1 (7.1)	4 (20.0)
COPD	2 (11.1)	0 (0.0)	1 (5.0)
Chronic renal failure	0 (0.0)	2 (14.3)	0 (0.0)
Liver failure	0 (0.0)	0 (0.0)	0 (0.0)
APACHE II	16 (10–21)	16 (13–23)	15 (11–20)
SOFA	8 (6–9)	10 (9–10)	7 (6–10)
Admission diagnosis			
Medical	18 (100.0)	11 (78.6)	7 (35.0)
Surgical	0 (0.0)	3 (21.4)	6 (30.0)
Trauma	0 (0.0)	0 (0.0)	7 (35.0)
Mean arterial pressure (mmHg)	92 (85–102)	80 (76–88) ^##^	85 (79–90) ^#^
White blood cell count (×10^3^ per μL)	9.9 (8.2–13.2)	12.6 (7.9–16.6)	14.7 (8.7–18.5)
Neutrophils (%)	88.2 (81.2–92.3)	86.3 (76.3–90.9)	89.6 (76.0–94.5)
Platelets (×10^3^ per μL)	266 (118–371)	220 (159–322)	233 (169–282)
PO_2_ (mmHg)	98.3 (76.6–117.8)	108.6 (85.6–145)	124.5 (92.2–167.0)
HCO_3_ (mEq/L)	22.9 (20.0–25.8)	22.1 (19.7–24.6)	21.4 (19.4–23.2)
PaO_2_/FiO_2_ (mmHg)	131 (95–177)	175 (100–209)	310 (202–381) ^#^
PCO_2_ (mmHg)	44.3 (40.8–50.8)	51.6 (38.7–56.0)	37.3 (34.5–42.3) ^# §^
Urea (mg/dL)	55 (47–67)	56 (33–87)	32 (26–43) ^# §^
Creatinine (mg/dL)	0.95 (0.68–1.33)	1.10 (0.68–2.88)	0.80 (0.60–0.98)
Total bilirubin (mg/dL)	0.69 (0.39–1.37)	0.55 (0.30–1.10)	0.63 (0.52–0.80)
LDH (U/L)	431 (449–615)	228 (215–489) ^##^	328 (255–425) ^#^
CRP (mg/dL)	9.8 (6.2–13.4)	17.3 (6.3–28.0)	6.5 (2.0–13.8) ^§^
Fibrinogen (mg/dL)	623 (527–718)	575 (424–763)	418 (255–535) ^### §^
pH	7.33 (7.29–7.36)	7.29 (7.24–7.38)	7.37 (7.31–7.42)
Mean vasopressor dose (mL/h)	5 (4–7)	12 (4–25) ^#^	12 (4–20)
Tidal volume (VT) (mL)	420 (408–463)	450 (380–500)	440 (405–480)
Respiratory rate (RR) (mechanical breaths/min)	27 (22–29)	24 (20–30)	22 (18–26) ^#^
Positive end-expiratory pressure (PEEP) (cmH_2_O)	12 (10–13)	11 (9–15)	8 (6–10) ^#### §^
Volume control (VC), N (%)	18 (100.0)	14 (100.0)	19 (95.0)
sICAM-1 (ng/mL)	630 (482–1014)	595 (490–1464)	447 (293–650) ^# §^
sVCAM-1 (ng/mL)	1488 (797–2166)	1395 (933–2414)	1147 (484–1703)
sE-selectin (ng/mL)	78.9 (60.4–108.8)	142.9 (107.3–226.5) ^##^	67.6 (57.1–117.9) ^§§^
vWf (pg/mL)	12313 (4581–15254)	7558 (4637–18020)	10632 (3723–17280)
ICU outcomes			
Length of ICU stay, days	28 (6–40)	25 (8–51)	14 (9–25)
Mortality, N (%)	5 (27.8)	4 (28.6)	2 (10.0)

Data are expressed as percentages of total related variable (%) and median (IQR) for skewed data. Measurements were taken upon admission to the intensive care unit. Norepinephrine was administered as a vasopressor. APACHE, acute physiology and chronic health evaluation; COPD, chronic obstructive pulmonary disorder; CRP, C-reactive protein; ICU, intensive care unit; LDH, lactate dehydrogenase; sICAM-1 = soluble intercellular adhesion molecule 1; SOFA, sequential organ failure assessment; sVCAM-1 = soluble vascular adhesion molecule 1; vWf, von Willebrand factor. Differences were tested by the chi-square test or by one-way ANOVA followed by Kruskal–Wallis. ^#^ *p* < 0.05, ^##^ *p* < 0.01, ^###^ *p* < 0.01, ^####^ *p* < 0.0001 compared to the COVID-19 ARDS group. ^§^ *p* < 0.05, ^§§^ *p* < 0.01 compared to the non-COVID-19 ARDS group.

**Table 2:** Demographics, clinical characteristics, and laboratory data of the COVID-19 ARDS survivors and non-survivors upon ICU admission.

Characteristics	Survivors	Non-Survivors	*p*-Value
Number of patients, N	12	5	
Age (years)	73 (63 79)	74 (65 78)	>0.99
Sex, N (%)			>0.99
Male	8 (66.7)	4 (80.0)	
Female	4 (33.3)	1 (20.0)	
Comorbidities, N (%)			>0.99
Hypertension	8 (66.7)	3 (60.0)	
Dyslipidemia	3 (25.0)	1 (20.0)	
Diabetes	3 (25.0)	0 (0.0)	
Coronary disease	3 (25.0)	1 (20.0)	
Cancer	2 (16.7)	1 (20.0)	
COPD	1 (8.3)	1 (20.0)	
APACHE II	15 (10–19)	20 (9–23)	0.51
SOFA	7 (4–9)	7 (6–12)	0.44
Mean arterial pressure (mmHg)	92 (88–101)	100 (81–118)	0.65
White blood cell count (×10^3^ per μL)	9.9 (9.0–12.5)	12.9 (4.9–24.9)	0.72
Neutrophils (%)	87.2 (81.5–91.3)	92.9 (73.9–93.5)	0.16
Platelets (×10^3^ per μL)	266 (136–386)	317 (54–605)	0.89
PCO_2_ (mmHg)	44.3 (41.4–48.3)	41.0 (36.3–65.0)	0.65
HCO_3_ (mEq/L)	21.9 (19.7–26.6)	25.2 (21.0–25.9)	0.57
PaO_2_/FiO_2_ (mmHg)	125 (83–193)	132 (107–195)	0.88
Glucose (mg/dL)	179 (139–220)	137 (120–188)	0.16
Urea (mg/dL)	52 (40–57)	65 (57–80)	0.06
Creatinine (mg/dL)	0.8 (0.6–1.3)	1.2 (1.0–1.3)	0.13
Total bilirubin (mg/dL)	0.7 (0.4–0.9)	1.5 (0.4–1.9)	0.51
LDH (U/L)	431 (344–589)	566 (372–4170)	0.51
CRP (mg/dL)	10.9 (6.5–14.1)	8.2 (5.4–24.8)	0.57
pH	7.32 (7.29–7.36)	7.35 (7.24–7.40)	0.51
Mean vasopressor dose (mL/h)	5 (3–5)	4 (3–31)	0.79
Tidal volume (VT) (mL)	430 (420–465)	410 (375–465)	0.51
Respiratory rate (RR) (mechanical breaths/min)	24 (21–28)	28 (28–30)	0.04 *
Positive end-expiratory pressure (PEEP) (cmH_2_O)	12.0 (10.3–14.8)	10.0 (8.0–12.5)	0.23
Dexamethasone administration, N (%)	10 (83.3)	4 (80.0)	>0.99
Day of death	N/A	6 (4–23)	

* *p* < 0.05. Data are expressed as percentages of total related variable (%) and median (IQR) for skewed data. Measurements were taken upon admission to the intensive care unit. Norepinephrine was administered as a vasopressor. APACHE, acute physiology and chronic health evaluation; COPD, chronic obstructive pulmonary disorder; CRP, C-reactive protein; LDH, lactate dehydrogenase; SOFA, sequential organ failure assessment. N/A not applicable.

## Data Availability

The raw data supporting the conclusions of this article will be made available by the authors upon request.
